# The lows of getting high: sentinel surveillance of injuries associated with cannabis and other substance use

**DOI:** 10.17269/s41997-018-0027-8

**Published:** 2018-02-26

**Authors:** Deepa P. Rao, Hanan Abramovici, Jennifer Crain, Minh T. Do, Steven McFaull, Wendy Thompson

**Affiliations:** 1 Surveillance and Epidemiology Division, Centre for Surveillance and Applied Research, Public Health Agency of Canada, Rm 707B1, 785 Carling Avenue, Ottawa, ON K1A 0K9 Canada; 2 Cannabis Legalization and Regulation Secretariat, Health Canada, Ottawa, ON Canada

**Keywords:** Cannabis, Substance use, Injury, Poisoning, Emergency department, Cannabis, Consommation de substances, Lésion, Intoxication, Service d’urgence

## Abstract

**Objectives:**

Cannabis is a widely used illicit substance that has been associated with acute injuries. This study seeks to provide near real-time injury estimates related to cannabis and other substance use from the electronic Canadian Hospitals Injury Reporting and Prevention Program (eCHIRPP) database.

**Methods:**

Data from the eCHIRPP database, years 2011 to 2016, were analyzed via data mining, descriptive, logistic regression, and sensitivity analyses. Drug use trends over time for cannabis and/or other substances (alcohol, illicit drugs, and medications) were assessed. Descriptive statistics (intent, external cause, and nature of injury) and proportionate injury ratios (PIR) associated with cannabis use are presented.

**Results:**

Cannabis use was observed in 184 cases/100,000 eCHIRPP cases, and related injuries were mostly identified as unintentional (66.8%). Poisoning (68.5%) and intoxication (69.4%) were the external cause and nature of injury most associated with these events, and hospitalization was recorded for 14.3% of cases. Per 100,000 eCHIRPP cases, cannabis was used alone in 72.4 cases, and in combination with alcohol, illicit drugs, or medications in 74.6 cases, 11.3 cases, and 7.9 cases, respectively. Relative to non-use, the PIR of hospitalization was not significant for cannabis-only users of either sex (males: PIR 1.0, 95% CI 0.6–1.7, females: PIR 0.9, 95% CI: 0.5–1.7).

**Conclusion:**

Cannabis use injuries are rare, but can occur when cannabis is used with or without other substances. As Canada considers legislative changes, our finding of cases related to unintentional injury, poisoning, and intoxication suggests areas that might benefit from health literacy efforts.

## Résumé

**Objectifs:**

Le cannabis, une substance illicite largement consommée, est associé à des blessures aiguës. Notre étude vise à présenter en quasi temps réel les blessures estimatives liées à la consommation de cannabis et d’autres substances d’après la base de données de la plateforme électronique du Système canadien hospitalier d’information et de recherche en prévention des traumatismes (eSCHIRPT).

**Méthode:**

Nous avons analysé les données de la base eSCHIRPT pour les années 2011 à 2016 au moyen de techniques de forage de données et d’analyses descriptives, de sensibilité et de régression logistique. Nous avons estimé les tendances de la consommation de cannabis avec ou sans autres substances (alcool, drogues et médicaments) au fil du temps. Nous présentons les statistiques descriptives (intention, cause externe et nature de la blessure) et les rapports proportionnels de blessures (RPB) associés à la consommation de cannabis.

**Résultats:**

La consommation de cannabis a été observée dans 184 pour 100,000 cas dans eSCHIRPT, et les blessures associées étaient principalement non intentionnelles (66,8%). L’empoisonnement (68,5%) et l’intoxication (69,4%) étaient la cause externe et la nature de la blessure les plus souvent associées à ces épisodes, et une hospitalization a été enregistrée dans 14,3% des cas. Pour 100,000 cas dans eSCHIRPT, le cannabis avait été consommé seul dans 72,4 cas, et en combinaison avec de l’alcool, de la drogue ou des médicaments dans 74,6, 11,3 et 7,9 cas, respectivement. Par rapport à la non-consommation, les RPB de l’hospitalization n’étaient pas significatifs pour les consommateurs et consommatrices de cannabis seul (hommes: RPB 1,0, IC de 95%: 0,6–1,7; femmes: RPB 0,9, IC de 95%: 0,5–1,7).

**Conclusion:**

Les blessures dues à la consommation de cannabis sont rares, mais elles peuvent se produire, que le cannabis soit consommé seul ou avec d’autres substances. Étant donné les modifications législatives envisagées au Canada, nos constatations sur les cas de blessures, d’empoisonnements et d’intoxications involontaires indiquent que des efforts pour renforcer l’information en matière de santé sur ces aspects pourraient être bénéfiques.

## Introduction

Cannabis (marijuana) is the most commonly used illicit drug worldwide (United Nations Office on Drugs and Crime 2016). Cannabis use is prevalent in Canada; in 2015, 12% of the population aged 15 and older reported past-year cannabis use (Health Canada 2015), up from 11% in 2013, and 41% reported having used it at least once in their lifetime (Health Canada 2013; Health Canada 2014). Among children in grades 7 to 12, cannabis had the highest prevalence of use after alcohol with nearly 17% reporting use in the year preceding the survey (Health Canada 2014). A majority of these children perceived regular cannabis use as associated with “great risk” (58%), while fewer (25%) identified irregular use as being of similar risk (Health Canada 2014). Among children younger than 12 years, there have been reports of unintentional exposures (Wang et al. 2013).

Acute ingestion of cannabis may result in time-limited cognitive, perceptual, and psychomotor perturbations which, when combined with physical activities such as driving, have been linked to mild, serious, or even potentially fatal physical injuries (e.g., bruises, fractures, concussions, death) (Hall 2015; Hall and Degenhardt 2014; Volkow et al. 2014). Even though studies have been inconsistent in suggesting a causal relationship between cannabis consumption and injury (Vitale and van de Mheen 2006; Elvik 2013; Mura et al. 2003), associations have nevertheless been reported with motor vehicle crashes (MVC) (Fischer et al. 2016; Ramaekers et al. 2004), interpersonal violence (Copeland et al. 2013), neighbourhood crime (de Looze et al. 2015), self-harm (Silins et al. 2014), and the use of other illicit drugs (Kaar et al. 2015).

Eight US states (Colorado, Washington, Oregon, Alaska, California, Maine, Massachusetts, and Nevada) and the District of Columbia (DC) have thus far legalized cannabis for non-medical purposes. Recent US public health surveillance data show increasing prevalence of cannabis use, mainly among adults, with increases in cannabis-associated fatal MVCs being reported in Colorado and Washington (Azofeifa et al. 2016; Reed 2016; Northwest High Intensity Drug Trafficking Area 2016). In advance of any similar proposed legislation to legalize cannabis for non-medical purposes in Canada (Task Force on Marijuana Legalization and Regulation 2016), this study aims to provide a baseline description of injuries related to cannabis and other substance use using data from the electronic Canadian Hospitals Injury Reporting and Prevention Program (eCHIRPP) database.

## Methods

### Data source

The eCHIRPP (Crain et al. 2016) is a dynamic web-based injury and poisoning surveillance system currently operating in 11 pediatric and 6 general emergency departments (ED) across Canada. It has been used to examine a number of health issues (Kang et al. 2013; McFaull et al. 2016; Health Surveillance and Epidemiology Division, Public Health Agency of Canada 2006). Patients’ accounts of pre-injury circumstances (narratives of “what went wrong”) are collected using the Injury Reporting form, a questionnaire completed during their visits to the ED. The attending physician, or other hospital staff, adds clinical data to the form and data coders extract other information found in patients’ narratives. Consequently, the eCHIRPP captures a broader assessment of an injury event, one that includes risk and protective factors and non-admitted cases, than other databases such as hospital administrative or mortality data alone (which often use less specific ICD codes). It captures injuries severe enough to require medical care, including both those that result in hospital admission as well as those that do not (Mackenzie and Pless 1999). eCHIRPP covers cases presenting to most major pediatric centres across Canada, but only select general hospitals. However, previous research has shown that it can represent general injury patterns among Canadian youth (Kang et al. 2013; Pickett et al. 2000). Records between April 1, 2011 and May 27, 2016 were extracted for the current analysis (*N* = 636,931 records).

### Definitions

Analyses are provided for all ages, children (ages 17 years and below), and adults (ages 18 years and above). Key terms used for narrative text mining are available on request.

#### Substances

The substance use variable code was screened for a listing of either ‘yes’ or ‘unknown’. Following this initial screen, other variable codes and/or narrative text were used to refine cases. Iterative data mining techniques were used to optimize queries of the narrative text. Criteria to identify specific substance use cases are detailed below:

- Cannabis use: (1) The substance ID and/or (2) narrative text contains terms like cannabis or marijuana. Criterion 1 was deemed sufficient since these would be based on clinical impressions, and cases where criterion 2 was positive but criterion 1 was negative were manually screened.
- Alcohol use: (1) The substance ID and/or (2) narrative text contains terms like alcohol. Criteria were assessed as described in section for cannabis use.
- Illicit drug use: (1) The substance ID and/or (2) narrative text contains terms like illicit drugs. Cases of cannabis use only were screened out. Criteria were assessed as described for cannabis use.
- Medication use: (1) The substance ID and/or (2) narrative text contains terms like medication (either over-the-counter or prescription-based). Criteria were assessed as described for cannabis use.

Combination of substances were analyzed as binary variables where instances of combined use were analyzed relative to individual or non-use (e.g., analyses of cases of cannabis use alongside alcohol use were made relative to cases of cannabis only, alcohol only, or no substance use).

#### Injury characteristics

##### Intent of injury event

Intent was examined to describe the external or environmental circumstances of the injury event. Intents involving police, emergency medical services, or other such professional staff are identified as cases where emergency response personnel (ERP) were involved. Intents were categorized based on intention of injury codes (IN codes) in combination with narrative text as follows: unintentional injury (10IN, 16IN, and key terms), physical assault and/or aggression (15IN and key terms), self-harm (11IN and key terms), ERP involvement (19IN and key terms), sexual assault (12IN and key terms), and maltreatment (13IN and 14IN).

##### External cause of injury

The external cause of injury variable code was used to describe the mechanism (external cause, EC codes) of injury, and key words were used to mine the narrative text as follows: poisoning (211EC, 210EC, 301EC, and key terms), fall (201EC and key terms), assault (4001EC, 400EC, and key terms), transport (100EC, 101EC, 102EC), and external agent (202EC, 203EC, 205EC, 209EC, 302EC, 305EC, 309EC, and key terms).

##### Nature of injury

Nature of injury variable codes (NI codes) were used to identify the type of injury among cases as follows: intoxication (50NI or key terms), external wound (10NI, 11NI, 20NI, 22NI), internal wound (24NI, 25NI, 26NI, 27NI, 52NI, 53NI, 60NI, 77NI), brain injury (41NI, 42NI, 43NI) or fracture, sprain, or strain (12NI, 13NI, 14NI, 15NI, 16NI, 17NI, 75NI, or key terms).

##### Severe injuries

These were defined as those injuries that required admission to hospital (treatment codes 700T, 800T, or 900T). Admission to hospital was used as a proxy for injury severity (dichotomized yes or no).

### Statistical analyses

Data mining syntax (PERL regular expressions (Zhang 2011)) was used to assess narrative text, and an analyst optimized the query language through an iterative process of comparing random samples of cases identified through data mining techniques with their corresponding narrative text. Manual resolution was conducted to ensure accuracy and precision of identified events. To assess the validity of the methodology chosen to identify cases of cannabis use, i.e., by either the use of substance ID and/or narrative coding, a sensitivity analysis was performed to estimate the sensitivity, specificity, positive predictive value (PPV), and negative predictive value (NPV) of each. Descriptive estimates of substance use relative to all eCHIRPP cases are reported as a proportion relative to 100,000 eCHIRPP cases. Injury characteristics related to cannabis use (intent, external cause, and nature of injury) are described as a proportion of all cannabis use cases. Among pediatric cases, age-adjusted proportionate injury ratio (PIR) estimates (Breslow and Day 1987) and 95% confidence intervals (CI) were calculated for each sex to examine the likelihood of severe injury based on substance use relative to severe injury among all other non-substance use cases presenting to the ED.

## Results

**Fig. 1:**
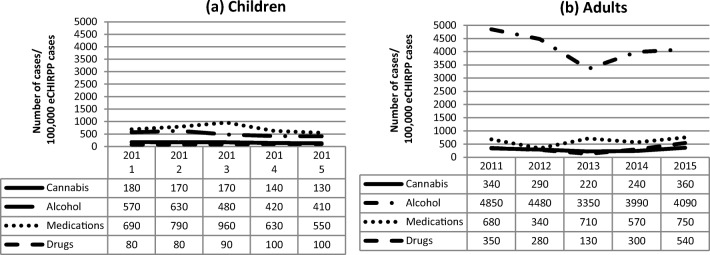
Time trend of substance use cases presenting to emergency departments among **(a)** children and **(b)** adults, eCHIRPP, 2011–2015. Records for 2016 were suppressed. eCHIRPP: electronic Canadian Hospitals Injury Reporting and Prevention Program

Between 2011 and 2016, 1170 cases of cannabis use cases were observed, representing an overall frequency of 184 cases/100,000 eCHIRPP cases. Among children, a total of 911 cases were observed for a frequency of 257 cases/100,000 eCHIRPP cases, and among adults, there were 258 cases for a frequency of 170 cases/100,000 eCHIRPP cases. For all ages, cannabis use was more frequent among males (57.1 versus 42.9% among females; children: 51.0% males and 49.0% females; adults: 79.1% males and 20.9% females) and among 15 to 19 years old (representing 58.3% of cannabis use cases, compared with 1.5% for those less than 10 years of age, 21.6% for 10 to 14 years, 9.5% for 20 to 29 years, 4.9% for 30 to 39 years, 2.7% for 40 to 49 years, and 1.6% for 50- to 64-year-old patients). Examining other substances, alcohol use was observed at a frequency of 1107 cases/100,000 eCHIRPP cases, medication use at 701/100,000 eCHIRPP cases, and illicit drug use at 130 cases/100,000 eCHIRPP cases. Time trends for these various substances are shown in Fig. 1.

A sensitivity analysis was conducted to assess the validity of our case identification method. The substance ID variable was found to be sensitive to 94.1% of cases, specific for 100.0% of cases, had a PPV of 100.0%, and an NPV of 99.9%. Narrative text mining was sensitive to 83.8% of cases, specific for 99.7% of cases, had a PPV of 37.3%, and an NPV of 100.0%. All 1101 cases identified based on the substance ID variable were kept as cannabis use cases, while another 69 cases were identified through mining of narrative codes. Narrative and substance ID codes were concordant in 77.9% of cases.

**Fig. 2:**
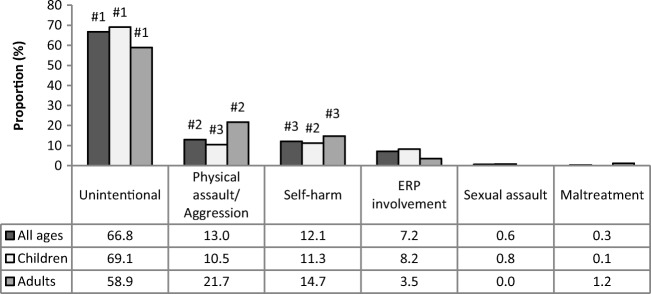
Distribution of intents of injury event among cannabis use-related cases, eCHIRPP, 2011–2016*. Numbers denote ranking of leading external causes listed within the database. Maltreatment refers to cases by a parent or caregiver, or by a spouse or partner. ERP: Emergency Response Personnel (police, emergency medical services, paramedic, etc.). eCHIRPP: electronic Canadian Hospitals Injury Reporting and Prevention Program. *Records entered on or before May 27, 2016

Examining cannabis cases, we observed that the intent, or external or environmental circumstances, of injury varied based on age. Unintentional injury was the leading intent of injury for all ages, and cases included a broad range of circumstances such as MVC, poisoning, and hallucinations. Self-harm was the second most frequent intent among children, and third among adults, while assault came in as the third and second most frequent intent, respectively (Fig. 2). Narrative text associated with cases of self-harm indicated thoughts or attempts at suicide from a variety of methods and included description of depression or feelings of sadness among the patient. For physical assault and/or aggression, cases involved description of altercations, for example getting into a fight with someone or being punched, or letting out aggression by punching an inanimate object. When examining the external cause, poisoning was the main route of injury for all ages. Cases of sexual assault involved cases where cannabis and/or other substances were in an individual’s system (either volitionally or because it was given to them) and they were sexually touched or raped. Finally, maltreatment cases involved aggressive behaviour or negligence by a partner or caregiver.

**Fig. 3:**
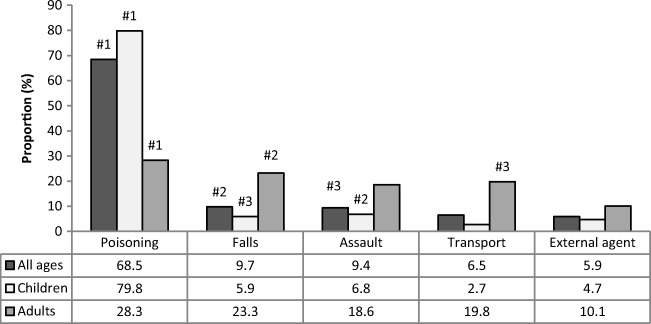
External cause of injury among cannabis use-related cases, eCHIRPP records, 2011–2016*. Numbers denote ranking of leading external causes listed within the database. External agent includes agents used for self-harm. eCHIRPP: electronic Canadian Hospitals Injury Reporting and Prevention Program. *Records entered on or before May 27, 2016

**Fig. 4:**
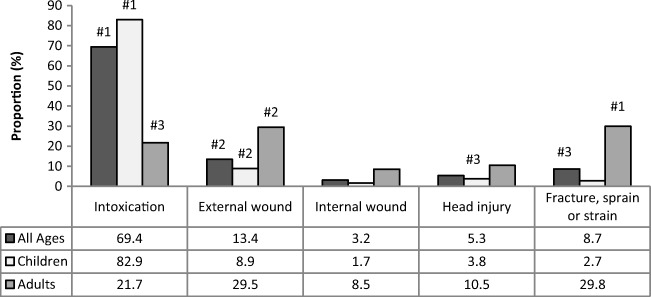
Nature of injury among cannabis use-related cases, eCHIRPP, 2011–2016* Numbers denote ranking of leading external causes listed within the database. eCHIRPP: electronic Canadian Hospitals Injury Reporting and Prevention Program. *Records entered on or before May 27, 2016

Falls accounted for a high proportion of external causes of injury for both age groups; however, assault was more frequent in child cases whereas transport was in adult cases. External causes involving external agents included circumstances that were both intentional, such as choosing to self-harm, or unintentional, such as bumping into an object (Fig. 3). With regards to the nature of injury, cases of intoxication were the highest ranked among children, while for adults, it was fractures, sprains, or strains. Of note, brain injuries were recorded for children, which included either minor head injury, concussion, or intracranial injury, and were the third highest ranking nature of injury (Fig. 4). Finally, per 100,000 eCHIRPP cases, cannabis use only (i.e., without any combination of substance) was observed among 72.4 cases, while cannabis in combination with alcohol was seen in 74.6 cases, with illicit drugs in 11.3 cases, and with medications in 7.9 cases (not including cases where 3 or more substances were combined).

Severe injury was observed in 14.3% of all cannabis use cases (10.1% among children and 29.1% among adults) and occurred due to the following external causes: poisoning (51.5%), transport (22.8%), external agent (9.6%), assault (8.4%), and falls (7.8%) (data not shown). Due to differences observed on the basis of sex, PIR estimates are presented for each sex and were examined only among pediatric cases (Table 1). The PIR of having a severe injury among male cannabis-only users, relative to severe injuries among males for all other injuries where no substance (cannabis, alcohol, drugs, medication) was used, was 1.0, 95% CI 0.6–1.7 and similarly among females was 0.9, 95% CI 0.5–1.7. When cannabis was used in combination with another substance, the PIRs became significant, except for cases of cannabis use in combination with alcohol among males. Combined medication and cannabis use among males, for instance, resulted in a significantly higher PIR of severe injury at 4.2, 95% CI 2.3–7.8 than observed among cannabis-only males, and similarly for females was 4.4, 95% CI 2.3–8.1 (Table 1). To provide comparison, the likelihood of a severe injury was examined independently for each of the other substances analyzed. We observed that for both sexes, the likelihood of severe injury was significantly higher in cases of medications only and illicit drugs only when compared with cannabis only (Table 1).

**Table 1:** Age-adjusted proportionate injury ratios by sex for severe injury based on substance use among children, eCHIRPP, 2011–2016

	Males		Females	
	PIR	95% CI	PIR	95% CI
Cannabis only	1.0	0.6–1.7	0.9	0.5–1.7
with alcohol	1.0	0.6–1.7	1.7	1.2–2.6
with drugs	1.9	1.0–3.9	2.4	1.1–5.0
with medications	4.2	2.3–7.8	4.4	2.3–8.1
Alcohol only	1.6	1.3–1.9	1.5	1.3–1.8
Illicit drugs only	2.1	1.4–3.1	2.7	2.0–3.7
Medications only	2.2	1.9–2.5	5.0	4.7–5.4

Severe injury defined as those cases where the individual was admitted to the hospital

*PIR* proportionate injury ratio, *CI* confidence interval, *eCHIRPP* electronic Canadian Hospitals Injury Reporting and Prevention Program

Records entered on or before May 27, 2016

## Discussion

The results of our study describe trends in substance use (cannabis, alcohol, medication, and illicit drugs) related ED presentations in the eCHIRPP database. Examining the four substances independently, the most frequent substance responsible for injury among children was medications, while for adults, it was alcohol; cannabis was the third and fourth, respectively. A variety of intents, external causes, and natures of injury associated with cannabis use were observed. Poisoning and intoxication, in particular, stood out as a leading external cause and nature of injury, respectively. The majority of injuries were unintentional in nature, although other intents included physical assault and self-harm. External causes of injury were mostly attributed to poisoning; however, falls and transport-related injuries were also observed. While intoxication once again was the main nature of injury, injuries such as fractures and open wounds were identified in the database. Finally, relative to all eCHIRPP cases not involving cannabis use, cannabis use was not significantly associated with hospitalization (i.e., severe injury), but its use in combination with illicit drugs and/or medications was. It is worth noting that of all eCHIRPP cases, when examining each substance independently, alcohol, illicit drug, and medication use, but not cannabis use, were significantly associated with hospitalization.

Our observed frequency of injuries related to substance use is similar with nationally reported substance use patterns for adults: alcohol use was reported among 77% of Canadians ages 15 and older, psychoactive pharmaceutical drugs at 22%, cannabis at 12%, and illicit drugs (excluding cannabis) at 2%. It appears that while cannabis use is known to be prevalent in Canada (Health Canada 2015), many intents, external causes, and injuries observed within eCHIRPP were related to overconsumption and/or toxic exposure to cannabis and its known or unknown combined substances. This may be indicative of health literacy regarding cannabis, i.e., that individuals may have had limited awareness regarding risks associated with cannabis use. According to a recent survey, more than a third (37.9%) of Canadians report that cannabis use should be permitted since they perceive it to not be a dangerous drug, with males agreeing to this statement significantly more than females (*p* < 0.001) (Health Canada 2006). Given this low perception of risk, it is also likely that many do not consider indirect harms associated with cannabis use such as falls or wounds, which accounted for a large number of injuries among adults. Even though harms from cannabis use are considered to be less likely than those associated with other psychoactive agents (Nutt et al. 2010), they are still present.

While differences in the proportion of cannabis use cases between the sexes were not observed in younger ages, adult males were more likely to be involved in an injury associated with cannabis use, which is consistent with usage patterns for each sex (Center for Behavioral Health Statistics and Quality 2015). Data from the US National Survey on Drug Use and Health show that males, aged 12 to 24, were more likely than females to list cannabis as their primary substance of abuse (Center for Behavioral Health Statistics and Quality 2014). We observed a higher proportion of injury cases among individuals aged 10 to 14 years; a demographic where recent findings suggest the importance of considering acute cannabis intoxication in cases of an altered level of consciousness (Murray et al. 2016).

Findings of injuries related to physical assault and self-harm are consistent with previous literature suggesting associations of cannabis use with the development of anxiety disorders, depression, suicide ideation, and interpersonal violence (Copeland et al. 2013). These instances may have been unintentional, likely related to a low awareness of harms (Health Canada 2006), but many were actually intentional and were consistent with behaviours to indicate distress (Doyle et al. 2017). The associations of cannabis use with intentional self-harm and with forms of assault also reveal important risks of harm and aggression, as described previously (Shorey et al. 2014; Hussey et al. 2006). ERP involvement was observed in a number of instances and includes cases where illegal activities were occurring, where individuals contacted the police regarding someone under the influence, or where police needed to detain someone for criminal behaviour. A recent Canadian study reported that crime rates in school neighbourhoods were indeed associated with cannabis use among adolescents (de Looze et al. 2015). Cannabis use is also a well-established risk factor for MVCs (Fischer et al. 2016), and this latter external cause of injury did appear among cases of injury among adults. Studies from British Columbia and Quebec suggest that between 12% and 14% of drivers involved in MVCs had cannabis in their system while driving (Senate Special Committee on Illegal Drugs 2002). A recent poll by the Canadian Automobile Association found that 26% of Canadians between the ages of 18 to 24 years believed that their driving under the influence of cannabis was either the same or better. These misconceptions may explain why almost two thirds of Canadians were concerned that roads would become more dangerous with the legalization of cannabis (Canadian Automobile Association 2016).

Among pediatric cases, severe injury was not found to be significantly associated with cases of cannabis use for either sex. Consistent with previous findings (Sewell et al. 2009), the use of cannabis in combination with alcohol resulted in greater severity of injury than with cannabis alone among males, though not significantly so. Recent reports suggest that prescription medication abuse is the fastest growing drug-related problem in the USA (Sarker et al. 2016), and results from our study also showed a significantly higher proportion of severe cases among users of medication and cannabis in combination, as well as medication alone, compared to cannabis alone. Similarly, our observation that severe injury among users of illicit drugs in combination with cannabis as more likely than with cannabis alone might reflect patterns describing users of these two substances in combination as having a reduced perception of the risks associated with them. Definitions used in this analysis for medications and for illicit drugs both contain agents that could be described as opioids and may therefore lend a perspective to previously described cases of the opioid crisis observed within the eCHIRPP dataset (Government of Canada 2017).

Information gathered from US States that have legalized cannabis for non-medical purposes (e.g., Colorado, Washington) report an increased number of ED visits and admissions to hospitals associated with possible cannabis exposure, increased calls to poison control centers mentioning human cannabis exposure, as well as increased numbers of fatalities among drivers positive for THC-only or THC-in-combination with alcohol or other drugs (Reed 2016; Northwest High Intensity Drug Trafficking Area 2016). Currently available sources of information regarding cannabis use and public health outcomes in Canada include national surveys, which mainly report on prevalence of use (Health Canada 2015; Health Canada 2013; Boak et al. 2015), and the Canadian Surveillance System for Poison Information, which provides information regarding toxoid exposures including those that do not present to EDs. The findings reported in this study help to shed light on external causes and natures of cannabis-related injuries based on ED data. Future assessments of eCHIRPP data against the current findings will assist in keeping track of how legalization may have affected observed cases.

### Strengths and limitations

A main strength of this study is the utility of the eCHIRPP database to capture and describe cases requiring medical care, but not necessitating hospital admission. The eCHIRPP is an active surveillance system where data collection is systematic, uses standardized coding, and has been ongoing for over 25 years. It is able to capture cases presenting to most major pediatric centres across Canada. As such, it is a useful data source for examining trends and detecting signals in the pediatric population.

Based on the nature and geography of participating centres, the eCHIRPP platform likely under-represents older teen, adult, aboriginal, rural, and fatal cases. Therefore, extrapolations for these subgroups are discouraged. Since this platform is also specific to ED presentations, it also does not capture mild or moderate cases that may not have sought care or that may have been dealt with through resources such as poison centres. The data do not distinguish between medical and non-medical cannabis use. Analyses were restricted to available variable codes, thereby restricting the level of detail available to describe cannabis use or injury descriptors. Since eCHIRPP is not population based, and since cannabis is not currently legal in Canada for non-medical use, under-reporting of injuries related to cannabis use is possible. Misclassification bias is a possibility given the lack of objectively collected substance use information. Definitions employed for data mining created a known bias towards accepting substance ID coding as confirmation of cannabis use; nevertheless, there was strong sensitivity and specificity of substance use and narrative codes for identifying cases. Since there was no known bias placed on examination of narrative codes, the specificity and NPV of using narrative codes point to the advantage of using this approach to correctly identify non-cases. A recent examination of the validity of self-reported substance use among emergency room populations and possible self-selection bias found that self-reported alcohol and substance use was actually preferable to other objective methods since the former provided more accurate information regarding actual use (Vitale et al. 2006).

## Conclusions

Cannabis use injuries were observed in the eCHIRPP database with a variety of intents, external causes, and natures of injuries. The current findings serve to describe a subsample of injuries that were significant enough to need medical attention but that did not always necessitate hospital admission. As Canada moves towards the legalization of cannabis for non-medical purposes, our observation of cases related to unintentional injury, poisoning, and intoxication suggests areas that might benefit from health literacy efforts.
